# Supercoiling, R-Loops, Replication and the Functions of Bacterial Type 1A Topoisomerases

**DOI:** 10.3390/genes11030249

**Published:** 2020-02-27

**Authors:** Julien Brochu, Émilie-Vlachos Breton, Marc Drolet

**Affiliations:** Département de Microbiologie, Infectiologie et Immunologie, Université de Montréal, Montréal, QC H3C 3J7, Canada; julien.brochu@umontreal.ca (J.B.); emilie.vlachos-breton@umontreal.ca (É.-V.B.)

**Keywords:** topoisomerases, *topA*, *topB*, topoisomerase I, topoisomerase III, R-loop, PriA, replication, *oriC*, supercoiling

## Abstract

Type 1A topoisomerases (topos) are the only topos that bind single-stranded DNA and the only ones found in all cells of the three domains of life. Two subfamilies, topo I and topo III, are present in bacteria. Topo I, found in all of them, relaxes negative supercoiling, while topo III acts as a decatenase in replication. However, recent results suggest that they can also act as back-up for each other. Because they are ubiquitous, type 1A enzymes are expected to be essential for cell viability. Single *topA* (topo I) and *topB* (topo III) null mutants of *Escherichia coli* are viable, but for *topA* only with compensatory mutations. Double *topA topB* null mutants were initially believed to be non-viable. However, in two independent studies, results of next generation sequencing (NGS) have recently shown that double *topA topB* null mutants of *Bacillus subtilis* and *E. coli* are viable when they carry *parC parE* gene amplifications. These genes encode the two subunits of topo IV, the main cellular decatenase. Here, we discuss the essential functions of bacterial type 1A topos in the context of this observation and new results showing their involvement in preventing unregulated replication from R-loops.

## 1. Introduction

DNA topoisomerases (topos) are essential enzymes that solve the topological problems associated with DNA transactions including replication, transcription and recombination. These nicking-closing enzymes cut either one (type I) or two (type II) DNA strands. Most topos are members of four different subfamilies; types IA, IB, IIA and IIB. To solve the topological problems, type 1A and type II enzymes use a strand passage mechanism, whereas type IB enzymes (also named swivelases), present in eukaryotic cells, use a rotation mechanism [1,2].

Type 1A topos are unique for three main reasons: They use ssDNA as substrates, they are the sole ubiquitous topos and many of them possess RNA topo activity [2,3,4]. The last two unique features strongly support the hypothesis that type 1A topos were present very early in evolution, i.e., at least in the last universal common ancestor (LUCA) and possibly in the RNA world [4]. Because of this, they are considered to be essential enzymes for viability; essentially meaning that their absence cannot be compensated by any type of mutation, including those modulating the activity of other topos.

Type 1A topos are classified into three subfamilies [4]; the two main families are topo I and topo III, for which the prototype enzymes are, respectively, *E. coli* topo I (*topA*)—the first topo to be discovered [5]—and *E. coli* topo III (*topB*). The third family is reverse gyrase that is only present in hyperthermophilic and some thermophilic organisms. Enzymes of the topo I subfamilies are found in all bacteria but not in archaea and eukaryotes, whereas topo III, the most ubiquitous, are present in most but not all bacteria and in all archaea and eukaryotes [4]. The current model for type 1A topo activity, recently supported by experimental evidence, involves a protein-mediated DNA gate mechanism for strand passage [6]. Although bacterial topo I and III can both relax negatively supercoiled DNA and decatenate DNA, they each have their preference for specific reactions. Topo III has a higher requirement for ssDNA than does topo I, and they mostly act as decatenases in replication and recombination, whereas topo I mostly acts to relax negative supercoiling during transcription [2,4]. The assumption that topo III has a higher requirement for ssDNA than topo I mostly stems from the observation that, as opposed to topo I which can relax DNA with a native supercoiling density (e.g., plasmid DNA extracted from *E. coli* cells), topo III cannot relax it unless the reaction is performed at 52 °C instead of 37 °C or an R-loop is present on the DNA template, two conditions that expose ssDNA regions [7,8]. Similarly, yeast top3 (the type 1A topo in yeast) and Drosophila topo IIIβ are also inefficient in relaxing DNA with a native supercoiling density, unless experimental conditions allowing ssDNA regions to be exposed are used [9,10].

Single-molecule analyses have shown that topo III relaxes DNA in fast processive runs but with long pauses between runs, whereas topo I relaxes DNA in slower processive runs but with much shorter pauses between the runs. The overall result is that topo I has a faster relaxation rate than topo III [11]. For decatenation, shorter pauses between decatenation cycles for topo III as compared to topo I can explain why topo III has a higher decatenation rate in single-molecule experiments [12]. More recent data indicate that differences in gate dynamics can explain the different substrate preferences of topo I and III [6]. The fast gate dynamics of topo I may facilitate efficient relaxation of negatively supercoiled DNA, whereas a slower gate-closing rate may facilitate capture of dsDNA and efficient decatenation by topo III.

*E. coli* cells lacking both type 1A topos were initially believed to be non-viable and were shown to form very long filaments and to possess abnormal nucleoid structures [13]. Furthermore, based on the observation that deleting *recA* partially corrected these phenotypes, it was proposed that, like eukaryotic topos IIIs, *E. coli* type 1A topos can resolve recombination intermediates [13]. Therefore, the resolution of recombination intermediates was viewed as the essential function of bacterial type 1A topos, and as one evolutionary conserved function of type 1A topos [13]. In this reaction RecQ, or its eukaryotic homologs Sgs1 and BLM act on the recombination intermediate to generate a hemicatenane, a structure that can only be resolved by a type 1A topo [2]. However, recent results have shown that both *E. coli* and *B. subtilis* cells lacking type 1A topos can survive when they overproduce topo IV [14,15]. Topo IV is a type II enzyme and therefore cannot resolve hemicatenanes. Topo IV is mostly involved in the resolution of topological problems related to replication [1]. Moreover, the interplay between topo IV and both topo I and III, respectively, related to supercoiling and replication, as has been described [16,17,18], and the beneficial effect of deleting *recA* in cells lacking type 1A topos can now been explained by the involvement of RecA in replication initiation from R-loops [14,19,20,21,22]. An R-loop is a three stranded nucleic acid structure in which the RNA is hybridized with the DNA template strand and the non-template DNA is single-stranded. Recent data have shown that both *E. coli* topo I and III can inhibit R-loop formation, which leads to the suggestion that this could be an evolutionary conserved function of type 1A topos [14]. In this review, we discuss the essential functions of type IA topos in bacteria in the context of these new results. Furthermore, we present a model in which bacterial type1A topos directly or indirectly prevent topological stress and genome instability due to over-replication.

## 2. Viability of Single topa Null Mutants and the Role of Topo I in Supercoiling Regulation

Bacterial topos I relax negative but not positive supercoiling because their substrate is ssDNA that is only present in the former. The first characterized *topA* null mutants had a deletion encompassing *cysB* and *topA* and were shown to grow almost as well as wild-type *E. coli* cells [23]. The growth of these *topA* null mutants was found to be possible owing to the presence of naturally acquired compensatory mutations in *gyrA* or *gyrB*, encoding the two subunits of gyrase [24]. These mutations were found to reduce the negative supercoiling level of the chromosome and the supercoiling activity of gyrase [25]. A model was therefore proposed in which the opposing enzymatic activities of gyrase and topo I set the optimal chromosomal negative supercoiling level for growth [24,25]. In the absence of *topA*, the negative supercoiling level is too high, and growth is inhibited. Some *topA* null cells manage to acquire compensatory gyrase mutations, allowing them to grow almost as well as wild-type cells.

In another study, the most frequent compensatory mutations for the absence of *topA* in *E. coli* were not found in *gyrA* or *gyrB* but in a chromosomal region including the *tolC* gene [26]. These compensatory mutations were in fact amplifications of this chromosomal region [27]. This region was later shown to include *parC* and *parE* genes encoding the two subunits of topo IV, and plasmids overproducing ParC and ParE were found to complement the growth defect of a *topA* null mutant [28]. Moreover, because topo IV could relax negatively supercoiled DNA, it was concluded that topo IV can compensate for the absence of topo I by relaxing the excess negative supercoiling introduced by gyrase. More recently, by using Next Generation Sequencing (NGS), an amplified region spanning 250 kb of DNA including *tolC*, *parE*, *parC* and several other genes was found in *E. coli* mutants lacking type 1A topos [14]. The fact that such an amplification did not accumulate in these mutants when they carried a plasmid that overproduced topo IV indicated that the amplification occurs when topo IV needs to be overproduced. In fact, topo IV is involved in the control of the level of chromosomal supercoiling in *E. coli* [16].

The amplification of the *parC parE* genes is a very frequent compensatory mutation for the absence of *topA*. Indeed, in a study in which a *gyrB*(Ts) allele was shown to compensate for the absence of *topA* at the gyrase semi-permissive temperature (37 °C), when *topA* null *gyrB*(Ts) cells were plated at 30 °C, the permissive temperature for gyrase, 90% of the cells managed to form colonies after one week of incubation [29]. Several colonies were analyzed and were all found to contain cells carrying an amplification of the chromosomal region encompassing *parC* and *parE* [29]. In another report addressing the viability of cells lacking topo I, it was concluded that *topA* null mutants could grow well without any compensatory mutations [30]. However, one of the strains constructed in this study was recently found to carry an amplification of *parC* and *parE* (Brochu and Drolet, unpublished). Therefore, *topA* null mutants of *E. coli* cannot form visible colonies, unless they accumulate compensatory mutations, a conclusion that was supported by the results of a recent study [31]. 

Interestingly, in *Shigella flexneri*, a species that is closely related to *E. coli*, *topA* null mutants can grow without compensatory mutations, albeit at a much slower rate than wild-type cells, and growth is not possible in some media [32]. It is possible that this *S. flexneri* gyrase is less active than the one in *E. coli,* and topo IV activity at its wild-type level is sufficient for *topA* null mutants to survive. Nevertheless, the introduction of a plasmid carrying the *S. flexneri parC* and *parE* genes was shown to correct *topA* null phenotypes of *S. flexneri* [33]. It is possible that *S. flexneri* is unable to amplify the region encompassing the *parC* and *parE* genes [32]. In a very recent study, *B. subtilis topA* null mutants that were able to form colonies were shown to carry *parC parE* amplifications [15]. However, no compensatory mutations were found in *gyrA* or *gyrB*. Thus, in bacteria with *parC* and *parE* genes, an appropriate level of topo IV allows *topA* null mutants to grow. However, some bacteria, such as *Mycobacterium tuberculosis* and *Helicobacter pylori*, do not possess topo IV. In these bacteria, topo I appears to be essential, as *topA* null mutants cannot be obtained [34,35].

**Figure 1:**
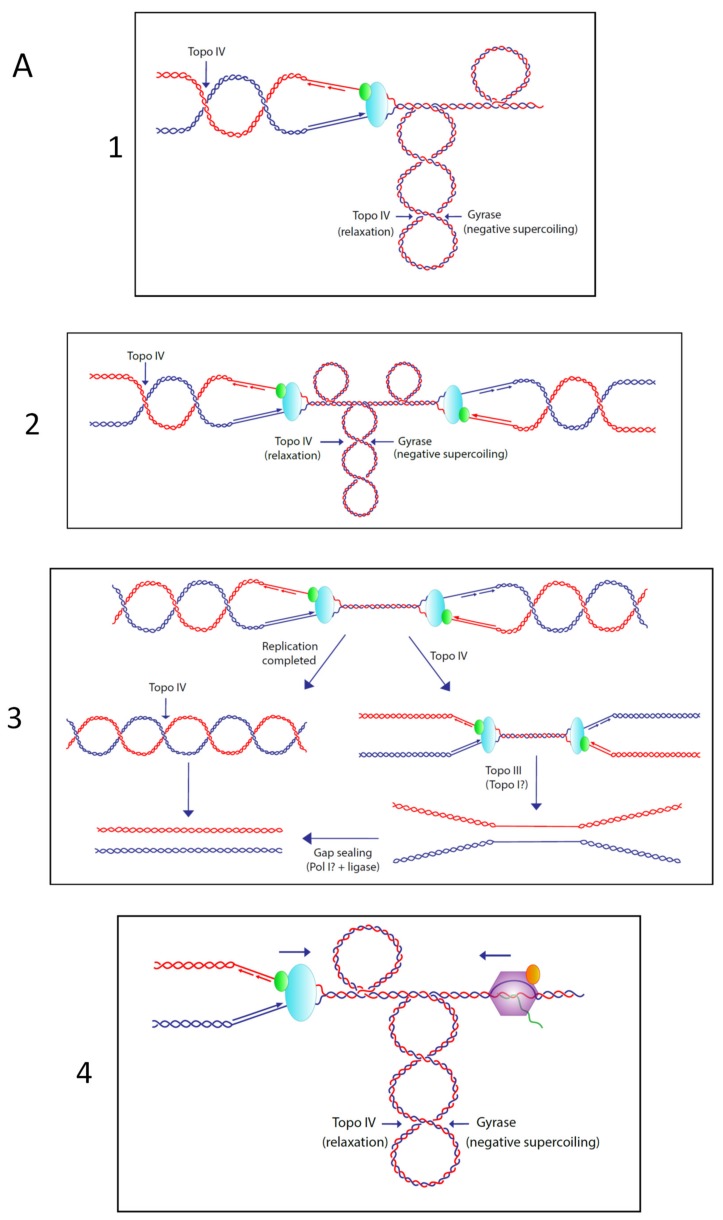

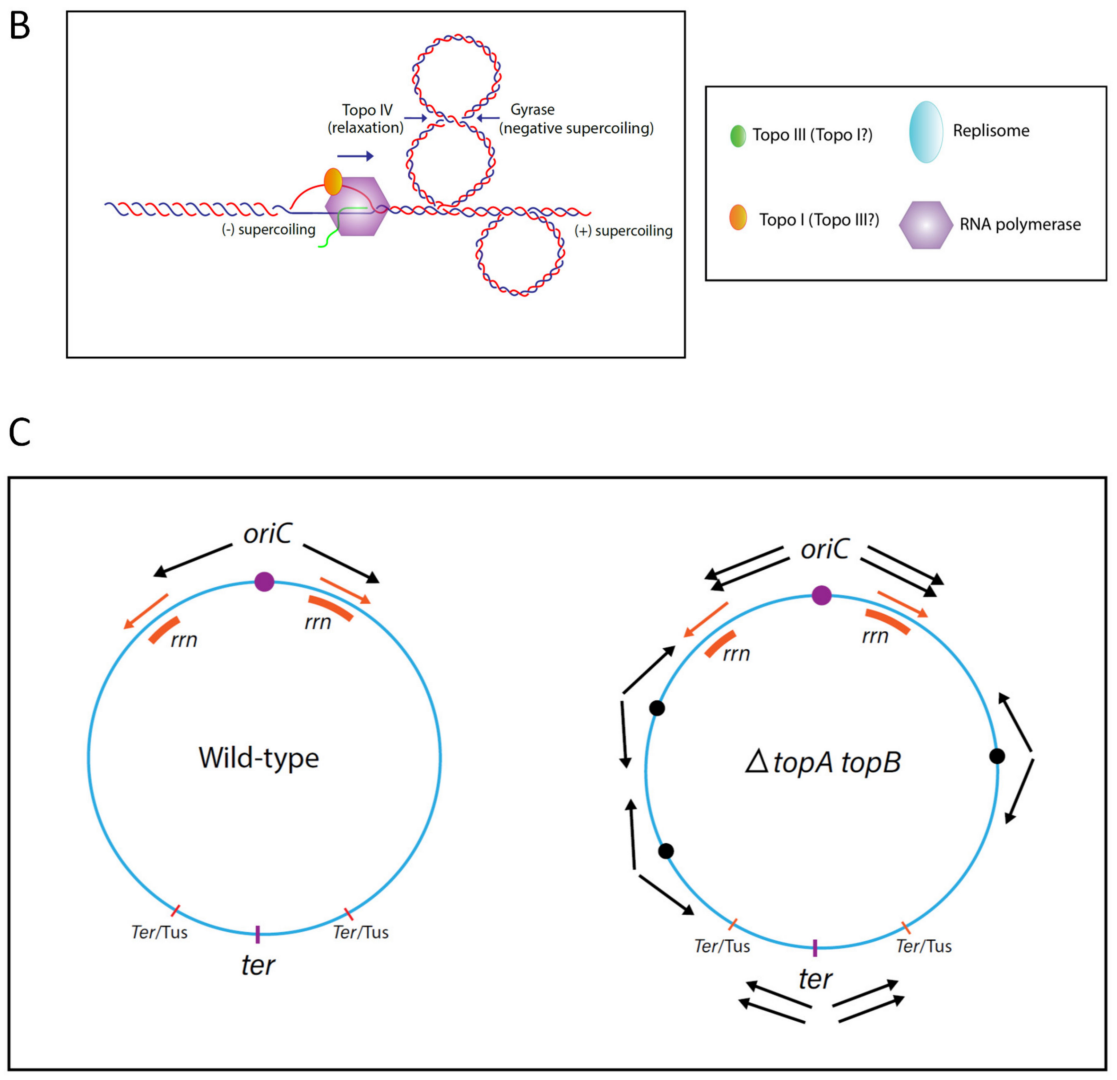
Model for the effects of deleting *topA* and *topB* on replication in *E. coli* cells. (**A**) Topological problems associated with replication elongation (1), replication termination (2 and 3) and head-on conflicts between replication and transcription (4). (**B**) Supercoiling during transcription. (**C**) In wild-type cells, bi-directional replication is initiated at *oriC* and is terminated when replication forks converge in the Ter region. Replication forks are trapped in the Ter region via the Ter/Tus barriers [106]. In *Escherichia coli* cells lacking type 1A topos, the topological problems illustrated in A are exacerbated because of over-replication from *oriC* and PriA-dependent replication initiation (e.g., R-loops) that takes place outside of *oriC*. Black and red arrows indicate the direction of, respectively, replication and transcription (*rrn* operons). The absence of type 1A topos activity during replication further aggravates the topological problems. See text for details.

High levels of local and transient supercoiling can be generated by transcription, as predicted by the twin-domain model [36]. Indeed, as transcription proceeds, negative and positive supercoiling are generated respectively behind and ahead of the moving RNA polymerase (RNAP). In bacteria, topo I acts behind RNAP to relax transcription-induced negative supercoiling, whereas gyrase acts in front to convert the positive supercoils (preferred substrates of gyrase) into negative ones [37] (Figure 1B). 

Recent data indicate that topos set the chromosomal supercoiling level in bacteria mostly by acting during transcription. Rovinskiy et al. used supercoiling sensors on the chromosome to monitor both supercoiling density and transcription elongation rates [38]. Their data support a model in which the average chromosomal supercoiling level is set by transcription elongation, with topo I and gyrase acting in concert. Gyrase cleavage sites on the chromosome of *E. coli* were recently mapped by using a ChIP-seq based approach [39]. The main factors governing the global distribution of gyrase molecules along the chromosome were shown to be transcription intensity and direction as well as binding preference for a degenerate motif [39]. 

ChIP-seq experiments have not yet been performed to map genome-wide topo I binding and cleavage sites on the chromosome of *E. coli*. Because topo I physically interacts with RNAP [40,41], these sites are expected to be largely determined by transcription. In fact, the full-length complete structure of topo I recently reported demonstrates how the C-terminal domain binds ssDNA to recognize the accumulation of negative supercoils and how this domain allows the rapid removal of transcription-induced negative supercoiling [42]. Interestingly, *E. coli topA* is under the control of four promoters that are recognized by different sigma factors, including the heat shock sigma factor σ32 and the general stress response sigma factor σS [43]. This indicates that topo I activity is always required during transcription, including when the cells are exposed to various stresses [43]. An interaction between RNAP and topo I, however involving different portions of the proteins as compared to *E. coli*, has also been observed for *M. tuberculosis* [44]. This may suggest that such an interaction has been evolutionarily conserved in bacteria. Perhaps the best evidence for the action of topo I and gyrase in transcription according to the twin-domain model has been obtained in *M. tuberculosis*, by using ChIP-seq approaches to map topo I, gyrase and RNAP binding sites [45]. Indeed, at a given transcriptionally active locus topo I and gyrase were found to be localized respectively behind and ahead of RNAP [45]. Thus, it is likely that topo I and gyrase act during transcription to set the chromosomal supercoiling level. 

## 3. The Role of Topo I in the Regulation of R-loop Formation in *E. coli*

One major consequence of excess negative supercoiling in *topA* null mutants is R-loop formation can lead to growth inhibition mediated by RNA degradation, and to unregulated replication that may cause genomic instability ([21,22,29,46,47,48] and see below). R-loop formation occurs during transcription and is promoted by negative supercoiling mainly because it requires the unwinding of the DNA strands to allow the annealing of the nascent RNA with the template strand. The involvement of negative supercoiling in R-loop formation is supported by the results of in vitro and in vivo experiments. In in vitro transcription reactions, R-loop formation is promoted when gyrase is present in sufficient excess over topo I [49]. Furthermore, R-loop formation in the presence of gyrase leads to hypernegative supercoiling of the plasmid DNA template [50]. In the presence of a sufficient amount of topo I or of RNase HI, an enzyme degrading the RNA moiety of R-loops, hypernegative supercoiling is inhibited [49,50]. Hypernegative supercoiling is best observed on plasmid DNA and is in part due to extensive unwinding (DNA relaxation) caused by R-loop elongation that is accompanied by gyrase activity introducing negative supercoils. When RNase HI is added after the reaction, hyper-negatively supercoiled DNA is revealed [48]. Furthermore, in vitro transcription of a hyper-negatively supercoiled plasmid DNA template leads to extensive and non-sequence specific R-loop formation [47]. Furthermore, work from Lieber’s group has shown that negative supercoiling by favoring the transient separation of the two DNA strands (DNA unwinding) promotes R-loop formation even in non-G-rich region that are normally very important for R-loop formation [51]. The interplay between DNA sequence and negative supercoiling in R-loop formation has been recently studied in more details [52].

The first evidence for R-loop formation in vivo was obtained by growing a conditional *topA gyrB*(Ts) mutant at 37 °C and then transferring the cells below 30 °C to reactivate gyrase. Growth inhibition and RNase HI-sensitive hypernegative supercoiling were observed as well as hyper-negatively supercoiled plasmid DNA carrying R-loops [29,53]. When RNase HI was overproduced, growth was restored and hypernegative supercoiling was inhibited [29,54]. Recently, R-loops were directly detected in this *topA* null mutant following the temperature downshift, by using the S9.6 antibodies that recognize RNA-DNA hybrids [14]. Interestingly, although a *topA* null strain carrying a naturally occurring *gyrB* compensatory mutation fails to significantly accumulate R-loops as measured by RNase HI-sensitive hypernegative supercoiling, a *gyrB^+^* derivative of this strain accumulates such hypernegative supercoiling and its growth is strongly stimulated by RNase HI overproduction [55]. Thus, the strong phenotypes related to R-loops in *topA* null mutants are mostly due to the supercoiling activity of gyrase in the context of the model of supercoiling regulation described above. However, evidence for local transcription-induced negative supercoiling directly promoting R-loop formation in the absence of topo I has also been shown [54,56]. It is likely that both mechanisms are required to reach the negative supercoiling level that triggers extensive R-loop formation in the absence of *topA*.

In P1 transduction experiments, unlike cells carrying a gyrase mutation or overproducing topo IV, cells overproducing RNase HI cannot readily accept a *topA* null allele [29,31]. This may suggest that R-loop formation is not the major cause of growth inhibition of *topA* null mutants. Alternatively, it is possible that upon topo I depletion, extensive R-loop formation occurs, and RNase HI overproduction rapidly leads to RNA degradation that inhibits growth. Interestingly, when *topA* null mutants are plated at 21 °C, a negative effect of RNase HI overproduction on the growth that correlates with the level of gyrase activity is observed [8,55]. More work is required to identify the major cause of growth inhibition of *topA* null mutants. Nevertheless, R-loop formation is clearly a major problem for *E. coli* cells lacking topo I. 

Further supporting the interplay between RNase HI and topo I in the metabolism of R-loops is the observation that double *topA rnhA* (RNase HI) null mutants can be non-viable [29,31,55]. Moreover, in transduction experiments, this non-viability is observed when a compensatory gyrase mutation, either a naturally acquired one or the *gyrB*(Ts) allele, is present [29,55]. Conditional *topA rnhA gyrB*(Ts) mutants were constructed in which the *rnhA* gene is under the control of an arabinose-inducible promoter on a plasmid [57]. Surprisingly, depletion of RNase HI due to the absence of arabinose lead to extensive DNA relaxation, even at 30 °C, the permissive temperature for gyrase [57]. This relaxation was in part due to the inhibition of the supercoiling activity of gyrase triggered by the absence of RNase HI, as shown by assays in crude cell extracts [57,58]. Whilst the DNA was extensively relaxed in the absence of arabinose following a temperature downshift from 37 to 30 °C, high levels of hypernegative supercoiling were observed in the presence of 0.005% of arabinose, a concentration of arabinose that presumably generated a wild-type level of RNase HI. In addition, the relaxation of hypernegative supercoiling was seen in the presence of 0.05% of arabinose that led to RNase HI overproduction [58]. Furthermore, in an independent experiment, DNA was found to be more relaxed in a *rnhA* mutant as compared to a wild-type strain (Egbe Bessong and Drolet, unpublished results). Taken together, these results suggest the existence of a regulatory circuit involving RNase HI and gyrase that may lead to the inhibition of the supercoiling activity of gyrase in the absence of RNase HI. Whether or not this regulatory circuit involves R-loops remains to be seen. Interestingly, in Arabidopsis chloroplasts, a physical interaction between RNase HI and gyrase was recently demonstrated and this interaction was shown to be important for the regulation of R-loop formation [59].

## 4. The Main Function of Topo III in *E. coli*

Topo III has a higher requirement for ssDNA than topo I. In vitro, *E. coli* topo III can relax DNA with a wild-type supercoiling density only at an elevated temperature [7], and in vivo topo III does not appear to play any role in supercoiling regulation [16,60]. However, in vitro topo III is a potent decatenase, provided that ssDNA regions (single-strand gaps in the nascent strands) are available for binding [7]. In an *oriC* plasmid DNA replication system and in the absence of gyrase, topo III can fully support bidirectional replication and decatenate the replicating daughter DNA molecules [61]. Moreover, in vitro, like topo IV, topo III is able to remove precatenanes [17,62]. Precatenanes are the result of the excess positive supercoiling ahead of the replication fork that migrates behind it, linking the two partially replicated sister chromosomes together [63] (Figure 1A, panel 1). Thus, topo III can act as a decatenase during replication, at least in vitro. 

In vivo, topo III overproduction is able to suppress both decatenation (*par* phenotype) and growth defects of *parC*(Ts) and *parE*(Ts) mutants at the non-permissive temperature [17]. *E. coli* cells lacking topo III are viable without compensatory mutations and they grow as well as wild-type cells [64]. However, double *ΔtopB parC*(Ts) and *ΔtopB parE*(Ts) mutants are non-viable at the permissive temperature [18]. Taken together, both the in vitro and in vivo results led the authors to propose that topo III likely acts at the replication fork to remove precatenanes during replication. Supporting this assumption is the recent finding that topo III is associated with the replication fork in vivo, likely via interactions with the single-stranded DNA-binding protein (SSB) and the DnaX complex of the Pol III holoenzyme [65]. Furthermore, the DnaX complex stimulates the ability of topo III to unlink catenated and precatenated DNA in vitro, and as shown by time-lapse microscopy, *ΔtopB* mutants have delayed and disorganized nucleoid segregation as compared to wild-type cells [65]. Thus, topo III acts at the replication fork to remove precatenanes during replication.

## 5. Viability of Double *topA topB* Null Mutants

The first paper about the characterization of *topA topB* null mutants led the authors to conclude that *E. coli* cells lacking type 1A topos were non-viable [13]. In the system used in this study, the *topA topB* null mutants (with naturally acquired compensatory gyrase mutations) carried a plasmid in which *topB* could be expressed from an arabinose inducible promoter, that could also be strongly repressed in the presence of glucose and the absence of arabinose. When cells were grown in minimal liquid media with glucose for several hours, they formed very long filaments fully packed with unsegregated and diffuse DNA [13]. Furthermore, when these cells were plated on minimal medium with arabinose and incubated for 24 h, a dramatic drop of plating efficiency was observed, as compared to *topA topB* null cells grown in arabinose-containing minimal liquid media. It was also reported that it is extremely difficult to obtain *topB* null transductants of *topA* null mutants, after 18 or 24 h of incubation, unless *topA* null mutants also carried a plasmid from which *topB* could be expressed [13,66]. However, in a subsequent paper, it was shown that double *topA topB* null transductants could be obtained after 48 h of incubation, thus leading the authors to conclude that cells lacking type 1A topos are viable if they acquire compensatory mutations [67]. However, the presence of such mutations in these strains has not been demonstrated. Using one of the *topA* null strains from this study along with a different one, another group later reported similar results, i.e., the appearance of *topA topB* null transductants after 48 h of incubation ([68], Usongo and Drolet unpublished results and see below). Thus, *topA topB* null mutants can be viable.

## 6. Topo III and RecQ: Lack of Experimental Evidence for the Presence of a “Toposome” in *E. coli*

Interestingly, in the initial study, deleting *recA* was shown to correct both the chromosome segregation and growth defects of a *topA topB* null mutant [13]. Since the *lexA3* mutations inhibiting the expression of the DNA damage-inducible SOS response did not correct the phenotypes of the *topA topB* null mutant, it was concluded that the recombinase function of RecA, but not its function in the induction of the SOS response, was involved in the phenotypes of *topA topB* null cells [13]. Furthermore, as stated in the discussion of their paper, the authors reported that depleting *topA* null cells for the RecQ helicase led to phenotypes identical to those of *topA topB* null cells (unpublished data in ref. [13]). Moreover, based on the observation that the RecQ helicase could stimulate the activity of topo III on different substrates in vitro [69,70] and the finding that a physical interaction takes place between top3and Sgs1 (RecQ homolog in yeast) in yeast [71], the authors proposed that *E. coli* topo III and RecQ form a “toposome” that can resolve RecA-mediated recombination intermediates [13]. In agreement with this conclusion, it was later reported that Drosophila and human topo IIIα, by physically interacting with BLM (RecQ homolog in Drosophila and humans) generate a complex that can resolve homologous recombination intermediates in vitro (Double Holliday Junctions, DHJs) [72,73,74]. In this reaction, the activity of RecQ-homologs on the recombination intermediates generates hemicatenanes, structures that can only be resolved by a type 1A enzyme. Thus, in the absence of type 1A topos, these structures cannot be resolved, and chromosome segregation is inhibited. Since that time, it has been generally accepted in the literature that an evolutionarily conserved major function of type 1A topos is the resolution of recombination intermediates [2]. 

However, although it is now well established that the resolution of recombination intermediates is a major function of topo III acting with RecQ-homologs in eukaryotic cells [2], it is much less clear in *E. coli*. Indeed, it has been recently shown that *topA recQ* null mutants, unlike *topA topB* ones, can be easily constructed and do not form very long filaments with unsegregated and diffuse DNA ([22] and Sutherland et al. unpublished results). Furthermore, topo III and RecQ do not physically interact in *E. coli* and it has been demonstrated that the complementation of *parC*(Ts) and *parE*(Ts) mutants at the non-permissive temperature by topo III overproduction does not require RecQ [17]. Lopez et al. reported that the synthetic lethality of double *ΔtopB parC*(Ts) and *ΔtopB parC*(Ts) could be partially suppressed by deleting *recQ* or *recA*, thus supporting the previous conclusion that topo III can act with RecQ to resolve recombination intermediates [60]. However, as mentioned by the authors, their finding that overproducing topo III could rescue the temperature sensitivity of a *ΔtopB ΔrecA parE*(Ts) strain was not compatible with a model in which topo III acts after RecA [60]. 

More recently, Perez-Cheeks et al. reported the results of their study about the phenotypes of *ΔtopB parC*(Ts) and *ΔtopB parE*(Ts) mutants [18]. However, instead of using the original *parC*(Ts) and *parE*(Ts) strains [28] that were used in the aforementioned paper [60], they reconstructed them. No effects of *recA* or *recQ* deletions on the growth of double *ΔtopB parC*(Ts) and *ΔtopB parC*(Ts) strains were observed [18]. Moreover, they found that *ΔtopB* cells were more sensitive to novobiocin, an inhibitor of topo IV and gyrase, than wild-type cells. This treatment of *ΔtopB* cells with novobiocin led to chromosome segregation defects that were shown to be due to the inhibition of topo IV, but not gyrase [18]. Their data therefore supported a role of topo III in replication, but not in recombination. As mentioned above, this conclusion was supported recently by the finding that topo III acts at the replication fork most likely to remove precatenanes [65]. Thus, a role for topo III with RecQ in the resolution of RecA-mediated recombination intermediates in *E. coli* is currently not well supported by experimental evidence. 

## 7. Topo IV Overproduction Allows *topA topB* Null Mutants of *E. coli* and *B. subtilis* to be Viable 

In a recent study, *topB* null transductants of *topA* null *gyrB*(Ts) strains could be obtained at the *topA* permissive temperature (37 °C). Quantitative whole genome sequencing was performed for one of the *topA topB* null mutants and revealed an amplification of a chromosomal region including the *parC* and *parE* genes [14]. The fact that this amplification was not detected when the *topB* null allele was transduced in a *topA* null *gyrB*(Ts) strain that carried a plasmid from which topo IV could be overproduced, indicated that the purpose of maintaining this amplification is indeed to overproduce topo IV. Based on the results of whole genome sequencing, a rapid and highly reproducible qPCR protocol was developed to reveal this amplification. In this protocol, appropriate oligos are used to determine the *qseC*/*lepA* ratio [14]. The *qseC* gene, close to *parC* and *parE*, is present in the amplified region whereas *lepA* is situated outside this region. On average, *topA topB* null cells carrying this amplification have a *qseC/lepA* ratio of 3 and more, whereas a ratio of close to 1 indicates the absence of this amplification [14]. So far, all the *topA topB* null strains tested in the *gyrB*(Ts) background were found to carry this amplification, whether they carried the *topA20*::Tn*10* or the Δ(*topA cysB*) allele and whether the *topB* null mutation was introduced before or after the *topA* null one [14]. 

When plated on LB medium at 37 °C the *topA* null *gyrB*(Ts) strains generate a mixture of small and large colonies, the smaller ones being more abundant [29]. The larger colonies are comprised of cells carrying an amplification of the chromosomal region including the *parC* and *parE genes* [29]. This result is best explained by the fact that the *gyrB*(Ts) mutation is not a naturally selected compensatory mutation for *topA null mutants*, and it is therefore probably not optimal to compensate for the lack of topo I. When measured, the *qseC/lepA* ratio is, on average, close to 1.5 to 2 [15] for the large colonies of *topA* null *gyrB*(Ts) cells [14]. Therefore, although *topA* null *gyrB*(Ts) mutants can grow without *parC parE* amplification, the presence of such an amplification confers a growth advantage. Thus, the fact that this amplification is always present in *topA topB* null *gyrB*(Ts) mutants, and moreover at a higher level as compared to *topA* null *gyrB*(Ts) strains, indicates that deleting *topB* in *topA* null cells exacerbates the *topA* null phenotypes. Interestingly, as stated above, to be viable *B. subtilis topA* null mutants need to overproduce topo IV [15]. These cells manage to overproduce topo IV via naturally selected mutations which are either amplifications of the *parCE* operon or mutations increasing the promoter strength of the *parCE* operon [15]. *B. subtilis topA topB* null mutants can be generated only when a *topB* null allele is introduced in a *B. subtilis topA* mutant overproducing topo IV [15]. Thus, both in *E. coli* and *B. subtilis*, overproducing topo IV allows *topA topB* null mutants to be viable. 

## 8. R-loop and RecA in Unregulated Replication in *topA topB* Null Mutants 

The *topA topB* null *gyrB(Ts)* mutants form very long filaments packed with unsegregated DNA [22], like the initially described *topA topB* null mutants [13], despite the fact that topo IV is overproduced due to the amplification of the *parC parE* genes. This phenotype is exacerbated and growth is strongly inhibited when *topA topB* null *gyrB(Ts)* cells are grown at 30 °C as compared to 37 °C [14]. The amount of topo IV molecules produced following *parC parE* genes amplification might be enough to allow *topA topB* null *gyrB*(Ts) cells to survive, but not enough to correct the growth and chromosome segregation phenotypes. However, as is the case for *topA* null *gyrB*(Ts) cells, RNase HI overproduction allows *topA topB gyrB*(Ts) null cells to grow much better at 30 °C [22]. Furthermore, RNase HI overproduction significantly corrects both the filamentation and chromosome segregation defects [22]. Interestingly, double *topA topB* null cells accumulate more R-loops that single *topA* null cells as shown by using the S9.6 antibodies [14]. Thus, once again these results strongly suggest that deleting *topB* exacerbates the phenotypes of *topA* null mutants.

Deleting *recA* improves the growth of both *topA* and *topA topB* null cells and overproducing RNase HI has no effect on the growth of these *recA^-^* cells [22]. Furthermore, like RNase HI overproduction, deleting *recA* significantly corrects both the filamentation and chromosome segregation defects of *topA topB* null cells [22]. These results suggest that RecA acts before RNase HI in the same pathway. Recent data indicate that this pathway is likely constitutive stable DNA replication (cSDR; [75,76]). This replication, first observed in *rnhA* mutants, still takes place in the presence of protein synthesis inhibitors (stable replication) long after replication from the normal origin of replication (*oriC*) is terminated (replication initiation from *oriC* is inhibited following protein synthesis inhibition). It is constitutive, as opposed to iSDR (inducible stable DNA replication) that requires the induction of the DNA damage SOS response [75]. This type of replication is believed to take place from R-loops that persist long enough in the absence of RNase HI to be used as primers by pol I. *rnhA* null cells can fully replicate their chromosome by using cSDR, as *oriC* and *dnaA* can be deleted in these cells [75]. Furthermore, RNase HI and topo I act as specificity factors in an in vitro reconstituted *oriC*-dependent replication system by preventing replication initiation outside of *oriC* [77,78]. In vivo, cSDR requires the PriA-dependent primosome that also includes PriB and DnaT proteins [22,75,76]. This replication system is also required for replication restart when a new replication fork needs to be re-assembled following replication arrest [79]. The recombinase function of RecA is also required for cSDR at the initiation step [19]. It has been hypothesized that RecA acts at the step of R-loop formation and this is supported by the observation that RecA can promote R-loop formation via an inverse strand exchange reaction in vitro [80,81]. 

More recently, cSDR has been detected in *topA gyrB*(Ts) and *topA topB gyrB*(Ts) cells, mostly at 30 °C as compared to 37 °C [21]. Moreover, as predicted since *topA topB* null cells accumulate more R-loops than *topA* null cells, a higher level of cSDR was detected in the former [21]. A marker frequency analysis (MFA) by NGS was then performed to map putative cSDR origins (previously named *oriKs*) in *topA topB* null mutants [14]. A major peak was detected in the chromosome terminus region (Ter), where replication initiated from *oriC* is normally terminated. A similar peak, although of lower intensity, was also detected in *rnhA* mutants [14,20,82]. Few other common peaks between *topA topB* and *rnhA* null cells outside the Ter region were also detected, but were of higher intensity in *rnhA* null cells [14]. The fact that overproducing RNase HI, deleting *recA* or mutating *dnaT* eliminated the Ter-located peak in *topA topB* null cells, strongly suggests that this peak corresponds to an *oriK* site, where replication is initiated from R-loops [14]. Furthermore, because all these genetic changes also significantly corrected the growth and chromosome segregation defects of *topA topB* null mutants, we can conclude that cSDR is a major problem for cells lacking type 1A topos [14].

Interestingly, a physical interaction between RecA and Topo I has been recently described [83]. RecA has been shown to stimulate the relaxation activity of topo I both in vitro and in vivo [84]. However, whether or not topo I can inhibit or counteract the strand invasion reaction mediated by RecA has not been tested. Yeast top3 (the type 1A topo of yeasts) can dissolve Rad51-mediated (eukaryotic RecA homolog) D-loops in vitro and in vivo [85,86,87]. Furthermore, dissolution by yeast top3 is possible only when D-loops have been made following the action of the cognate recombinase, i.e., yeast Rad51, but not RecA or human Rad51 [86]. Although this result strongly suggests that yeast top3 and Rad51 interact with each other, such an interaction has not yet been demonstrated. Yeast Rad51 can promote R-loop formation in vivo [88]. One possibility is that the interaction between topo I and RecA may lead to D-loop and R-loop dissolution, thus preventing the assembly of PriA-dependent primosomes and the ensuing unregulated replication. 

## 9. Topo III: A Specific Role in the Regulation of R-loop Formation or Simply a Back-up for Topo I? 

As shown above, deleting *topB* exacerbates phenotypes of *topA* null cells, including a requirement for high levels of topo IV activity, R-loop formation and cSDR activity. This may suggest that topo III acts as a back-up for topo I, i.e., that topo III performs topo I functions in its absence. This interpretation is supported by the observation that no cSDR is detected in single *topB* null mutants and that whilst deleting *topA* makes *rnhA* mutants non-viable [29,31,55], deleting *topB* has no effects on the growth and cell morphology of *rnhA* mutants [21]. Acting as a back-up for *topA* would mean that topo III is able to perform function(s) of topo I, leading to the inhibition of R-loop formation and cSDR. As stated above, the main function of topo I is the relaxation of transcription-induced negative supercoiling. In fact, in an in vitro transcription system, topo III is able to relax transcription-induced negative supercoiling, though less efficiently than topo I [8]. Further works need to be done to see if deleting *topB* in a *topA* null mutant leads to a higher level of hyper-negatively supercoiled DNA. 

Interestingly, in vitro, an R-loop is a hot-spot for the relaxation activity of both topo I and III. In agreement with the higher requirement for a stable ssDNA region for topo III as compared to topo I, topo III is better than topo I in relaxing an R-looped plasmid DNA [8,49]. Moreover, the strong RNA topo activity of topo III might be involved in the removal of R-loops [3]. More work is needed to better understand the involvement of topo III in the metabolism of R-loops. Nevertheless, these data suggest that very early in evolution, before the occurrence of gene duplications leading to topo I and topo III, the main function of type 1A topos might have been to control R-loop formation to avoid over-replication. Interestingly, results supporting the involvement of human TOP3β in the inhibition of R-loop formation to prevent genomic instability have been recently presented [89]. 

## 10. Topo I in the Regulation of Replication from *oriC*

Replication initiation from *oriC* is initiated by the binding of DnaA proteins, followed by DNA unwinding and the loading of DnaB helicases via DnaC [90,91]. Negative supercoiling is required both in vitro and in vivo for DNA unwinding at *oriC* [92,93,94]. Inhibition of DNA gyrase in vivo leads to the inhibition of replication from *oriC*. In vitro, topo I can inhibit replication from *oriC* [61] and in vivo *topA* mutants can suppress the temperature sensitivity of *dnaA*(Ts) mutants [95]. Replication is still initiated from *oriC* in *topA dnaA*(Ts) mutants as it is not possible to delete *oriC* or the *dnaA* gene [95]. Presumably, the high level of negative supercoiling at *oriC* in the absence of topo I can compensate for the low level of DnaA activity in *dnaA*(Ts) mutants, at the non-permissive temperature. In fact, recent results suggest that topo I regulates replication initiation at *oriC* by reducing transcription-induced negative supercoiling in this region [96]. Furthermore, replication from *oriC* is over-initiated and asynchronous with the cell cycle in a *topA* null mutant [22,68]. 

In a suppressor screen (Tn*5* transposon) of the growth defect of a *topA rnhA* null mutant, genes involved in cSDR (including *dnaT*) and replication in general were isolated, thus indicating that this double mutant suffered from over-replication [22,58]. One of the suppressors had the Tn*5* inserted close to the middle of the *oriC* region [22]. This *oriC* mutation was shown to be non-viable in wild-type or *gyrB*(Ts) strains but was perfectly viable in the *topA* null *gyrB*(Ts) strain [22]. This mutation exacerbated the asynchrony phenotype of replication initiation at *oriC* in the *topA* null mutant but corrected the over-replication phenotype at *oriC* [22]. Thus, topo I plays a major role in the control of both PriA- (R-loops and maybe D-loops) and *oriC*-dependent replication initiation.

## 11. A Major Problems of *E. coli* Cells Lacking Type1A Topos: Over-Replication Leading to Topological Stress and Genomic Instability

The phenotypes of *topA topB* null cells can best be explained in the context of two observations: Deleting *recA* significantly corrects the phenotypes of *topA topB* null cells and an appropriate level of topo IV activity is critical for the viability of such cells. As stated above, recent data indicate that the effect of deleting *recA* cannot be explained in the frame of the last step of the homologous recombination reaction, i.e., the resolution of recombination intermediates as previously thought, but in the context of the earlier step of strand invasion, to form R-loops and maybe D-loops. Indeed, the positive effect of RNase HI overproduction is not observed in *topA topB* null cells in which the *recA* gene has been deleted, and RNase HI overproduction or *recA* deletion inhibits cSDR in these *topA topB* null cells. As stated earlier, RecA is involved in the initiation step of cSDR and can promote R-loop formation in vitro. Because deleting *recA* or overproducing RNase HI significantly corrects the growth defect of *topA topB* null cells, we can conclude that cSDR is a major problem for cells lacking type 1A topos. This is also supported by the observation that a *dnaT* mutation, affecting a component of the PriA-dependent primosome, inhibits cSDR and partially corrects the phenotypes of *topA topB* null cells. Top3 in yeast can dissolve yeast Rad51-mediated D-loops in vitro [86] and evidence for such a reaction also taking place in vivo has been presented [85,87]. In this context, the observation that RecA and topo I physically and functionally interact in *E. coli* may suggest that deleting *topA* could also promote PriA-dependent unregulated replication from D-loops. Moreover, since RecA and Rad51 can both promote R-loop formation, it is possible that an R-loop dissolution reaction involving a type 1A topo is also occurring in the cell. 

Topo IV shares common functions with both topo I and topo III. Indeed, like topo I, topo IV is involved in the relaxation of negative supercoiling and, like topo III, topo IV can remove precatenanes that form as a result of replication. For DNA relaxation, topo I plays the major role, whereas for decatenation, topo IV is the major cellular enzyme. Thus, the positive effect of overproducing topo IV in *topA topB* null cells could be mediated through DNA relaxation that would reduce R-loop formation and therefore cSDR, and/or through an increase in decatenation activity required because of over-replication. 

Interestingly, it has been recently shown in yeast that the role of limiting replication initiation via the checkpoint–kinase response induced by DNA damage, is to prevent topological stress that leads to increased DNA catenation, followed by DNA damage and chromosome loss [97]. Topological stress is also observed in non-stressed S-phase cells when too many replication origins are simultaneously activated. In the absence of type1A topos, replication from *oriC* is asynchronous with the cell cycle and over-initiated, and unregulated PriA-dependent replication from R-loops (and maybe D-loops) is activated. As a result, an elevated number of replication forks are simultaneously traveling on the chromosome, with some of them moving in opposite directions. DNA gyrase, especially in cells carrying a compensatory *gyrA* or *gyrB* mutation, may be unable to deal with the unusually high level of positive supercoiling generated by this over-replication. As a result, a high number of precatenanes is generated, thus requiring high levels of topo III and topo IV activity to remove them (Figure 1A). Since topo III is absent, topo IV activity can be rapidly saturated, and its overproduction would then be required for cell survival. Furthermore, when replication forks converge in the chromosomal Ter region and also outside Ter in *topA topB* null cells, the space on the DNA template becomes too small to accommodate the binding of DNA gyrase. In this situation, two alternative pathways, one involving topo IV (the major pathway) and the other involving topo III, can be used to complete replication while allowing the last DNA intertwines to be removed to fully decatenate the chromosomes [21,98] (Figure 1A, panels 2 and 3). Once again, in this situation, topo IV might need to be overproduced in *topA topB* null cells. 

Head-on conflicts between replication forks and heavily-transcribed *rrn* (rRNA) operons, that are avoided when replication is initiated from *oriC*, can occur in *topA topB* null cells and generate topological problems similar to those encountered when replication forks converge (Figure 1A, panel 4). Indeed, *rrn* operon transcription is co-directional with replication forks initiated at *oriC*. However, when replication is initiated at other sites on the chromosome, as is the case for R-loop-dependent replication, head-on conflicts may occur. Recent data support the involvement of yeast top2 and top3 in the resolution of topological problems related to conflicts between replication and rDNA transcription [99]. In *E. coli*, as it is the case for replication fork convergence, topo IV and topo III might be involved in the resolution of such topological problems. Therefore, overproducing topo IV could be required to deal with these problems in *topA topB* null cells. As is the case for topo III acting as a back-up for topo I, it is also possible that topo I acts as a back-up for topo III when it is absent. In this case, the decatenation phenotype of topo III would be strongly exacerbated in the absence of topo I. 

In *E. coli* cells in which the orientation of *rrn* operon has been inverted, RecBCD is vital for the processing of forks stalled due to head-on conflicts [100]. Indeed, in the absence of RecBCD, lethal dsDNA breaks are generated at stalled replication forks due to head-on conflicts with *rrn* operon transcription [100]. Because of the occurrence of such head-on conflicts due to R-loop-dependent replication, *rnhA recB* cells are non-viable [20]. *topA* null *gyrB*(Ts) cells lacking *recB* are barely viable [22], thus supporting the occurrence of potentially lethal conflicts due to R-loop-dependent replication in these cells.

Although topo IV can contribute to supercoiling regulation by directly relaxing negatively supercoiled DNA [16], it may also act by substituting for gyrase in removing the positive supercoiling generated by replication and transcription. This could occur mostly when topo IV is overproduced. By doing so, the positive supercoiling is relaxed and not converted into negative supercoiling as normally done by gyrase. In this situation, topo IV would also reduce the accumulation of precatenanes during replication. In the study of Rovinskiy et al. in which supercoiling sensors on the chromosome were used to monitor both supercoiling density and transcription elongation rates, data were presented suggesting that topo IV can also act in front of RNAP to remove positive supercoils [38]. In another study, ChIP-seq protocols were used to map genome-wide topo IV binding and cleavage sites [101]. Although replication was found to be the main factor influencing binding and cleavage site selection, transcription was also shown to be important, as a short treatment with rifampicin abolished topo IV cleavage at tested sites [101]. It is also important to mention that topo IV relaxes positive supercoiling at a 20-fold faster rate than negative supercoils [102].

## 12. Conclusions

In the past, phenotypes related to *topA* mutations have been mostly attributed to the effect of supercoiling on gene expression. Although supercoiling regulation clearly plays an important role in bacterial gene expression [103], recent data indicate that one major function of topo I is related to replication. This is also the case for the other type1A enzyme, topo III. Thus, bacterial type 1A enzymes are required to inhibit inappropriate replication initiation events and to deal with topological problems related to replication. Failure of type 1A topos to act in replication may lead to genomic instability and cell death. Rapidly growing bacteria possess two type 1A topos, plus topo IV and gyrase. The presence of topo IV and a specific type 1A enzyme (topo III) to act as decatenase enzymes during replication might be required to support a high growth rate. Rapid growth requires a high level of transcription that may exacerbate the topological problems related to replication by promoting R-loop formation and replication-transcription conflicts. The slow growing bacteria *M. tuberculosis* possesses only two topos; topo I and gyrase. In this case, topo I enzymatic activity may be optimal not only for supercoiling regulation, but also for decatenation during replication. In fact, recent data suggest an important role of *M. tuberculosis* topo I in chromosome segregation [104]. More experiments are required to better understand the role(s) of the ubiquitous type 1A topos and their importance in the maintenance of genomic stability. Interestingly, recent data show that point mutations in *E. coli topA* can increase the rate of a unique mutation spectrum that enhances the emergence of antibiotic resistance [105]. 

## References

[1] Vos S.M., Tretter E.M., Schmidt B.H., Berger J.M. (2011). All tangled up: How cells direct, manage and exploit topoisomerase function. Nat. Rev. Mol. Cell. Biol..

[2] Chen S.H., Chan N.L., Hsieh T.S. (2013). New mechanistic and functional insights into DNA topoisomerases. Annu. Rev. Biochem..

[3] Ahmad M., Xue Y., Lee S.K., Martindale J.L., Shen W., Li W., Zou S., Ciaramella M., Debat H., Nadal M. (2016). RNA topoisomerase is prevalent in all domains of life and associates with polyribosomes in animals. Nucleic Acids Res..

[4] Garnier F., Debat H., Nadal M. (2018). Type IA DNA Topoisomerases: A Universal Core and Multiple Activities. Methods Mol. Biol..

[5] Wang J.C. (1971). Interaction between DNA and an *Escherichia coli* protein omega. J. Mol. Biol..

[6] Mills M., Tse-Dinh Y.C., Neuman K.C. (2018). Direct observation of topoisomerase IA gate dynamics. Nat. Struct. Mol. Biol..

[7] DiGate R.J., Marians K.J. (1988). Identification of a potent decatenating enzyme from *Escherichia coli*. J. Biol. Chem..

[8] Broccoli S., Phoenix P., Drolet M. (2000). Isolation of the topB gene encoding DNA topoisomerase III as a multicopy suppressor of topA null mutations in *Escherichia coli*. Mol. Microbiol..

[9] Kim R.A., Wang J.C. (1992). Identification of the yeast TOP3 gene product as a single strand-specific DNA topoisomerase. J. Biol. Chem..

[10] Wilson T.M., Chen A.D., Hsieh T. (2000). Cloning and characterization of Drosophila topoisomerase IIIbeta. Relaxation of hypernegatively supercoiled DNA. J. Biol. Chem..

[11] Terekhova K., Gunn K.H., Marko J.F., Mondragon A. (2012). Bacterial topoisomerase I and topoisomerase III relax supercoiled DNA via distinct pathways. Nucleic Acids Res..

[12] Terekhova K., Marko J.F., Mondragon A. (2014). Single-molecule analysis uncovers the difference between the kinetics of DNA decatenation by bacterial topoisomerases I and III. Nucleic Acids Res..

[13] Zhu Q., Pongpech P., DiGate R.J. (2001). Type I topoisomerase activity is required for proper chromosomal segregation in *Escherichia coli*. Proc. Natl. Acad. Sci. USA.

[14] Brochu J., Vlachos-Breton E., Sutherland S., Martel M., Drolet M. (2018). Topoisomerases I and III inhibit R-loop formation to prevent unregulated replication in the chromosomal Ter region of *Escherichia coli*. PLoS Genet..

[15] Reuss D.R., Fasshauer P., Mroch P.J., Ul-Haq I., Koo B.M., Pohlein A., Gross C.A., Daniel R., Brantl S., Stulke J. (2019). Topoisomerase IV can functionally replace all type 1A topoisomerases in Bacillus subtilis. Nucleic Acids Res..

[16] Zechiedrich E.L., Khodursky A.B., Bachellier S., Schneider R., Chen D., Lilley D.M., Cozzarelli N.R. (2000). Roles of topoisomerases in maintaining steady-state DNA supercoiling in *Escherichia coli*. J. Biol. Chem..

[17] Nurse P., Levine C., Hassing H., Marians K.J. (2003). Topoisomerase III can serve as the cellular decatenase in *Escherichia coli*. J. Biol. Chem..

[18] Perez-Cheeks B.A., Lee C., Hayama R., Marians K.J. (2012). A role for topoisomerase III in *Escherichia coli* chromosome segregation. Mol. Microbiol..

[19] Kogoma T., Skarstad K., Boye E., von Meyenburg K., Steen H.B. (1985). RecA protein acts at the initiation of stable DNA replication in rnh mutants of *Escherichia coli* K-12. J. Bacteriol..

[20] Dimude J.U., Stockum A., Midgley-Smith S.L., Upton A.L., Foster H.A., Khan A., Saunders N.J., Retkute R., Rudolph C.J. (2015). The Consequences of Replicating in the Wrong Orientation: Bacterial Chromosome Duplication without an Active Replication Origin. mBio.

[21] Martel M., Balleydier A., Sauriol A., Drolet M. (2015). Constitutive stable DNA replication in *Escherichia coli* cells lacking type 1A topoisomerase activity. DNA Repair (Amst).

[22] Usongo V., Drolet M. (2014). Roles of type 1A topoisomerases in genome maintenance in *Escherichia coli*. PLoS Genet..

[23] Sternglanz R., DiNardo S., Voelkel K.A., Nishimura Y., Hirota Y., Becherer K., Zumstein L., Wang J.C. (1981). Mutations in the gene coding for *Escherichia coli* DNA topoisomerase I affect transcription and transposition. Proc. Natl. Acad. Sci. USA.

[24] DiNardo S., Voelkel K.A., Sternglanz R., Reynolds A.E., Wright A. (1982). *Escherichia coli* DNA topoisomerase I mutants have compensatory mutations in DNA gyrase genes. Cell.

[25] Pruss G.J., Manes S.H., Drlica K. (1982). *Escherichia coli* DNA topoisomerase I mutants: Increased supercoiling is corrected by mutations near gyrase genes. Cell.

[26] Raji A., Zabel D.J., Laufer C.S., Depew R.E. (1985). Genetic analysis of mutations that compensate for loss of *Escherichia coli* DNA topoisomerase I. J. Bacteriol..

[27] Dorman C.J., Lynch A.S., Ni Bhriain N., Higgins C.F. (1989). DNA supercoiling in *Escherichia coli*: TopA mutations can be suppressed by DNA amplifications involving the tolC locus. Mol. Microbiol..

[28] Kato J., Nishimura Y., Imamura R., Niki H., Hiraga S., Suzuki H. (1990). New topoisomerase essential for chromosome segregation in *E. coli*. Cell.

[29] Drolet M., Phoenix P., Menzel R., Masse E., Liu L.F., Crouch R.J. (1995). Overexpression of RNase H partially complements the growth defect of an *Escherichia coli* delta topA mutant: R-loop formation is a major problem in the absence of DNA topoisomerase I. Proc. Natl. Acad. Sci. USA.

[30] Stupina V.A., Wang J.C. (2005). Viability of *Escherichia coli* topA mutants lacking DNA topoisomerase I. J. Biol. Chem..

[31] Stockum A., Lloyd R.G., Rudolph C.J. (2012). On the viability of *Escherichia coli* cells lacking DNA topoisomerase I. BMC Microbiol..

[32] Ni Bhriain N., Dorman C.J. (1993). Isolation and characterization of a topA mutant of Shigella flexneri. Mol. Microbiol..

[33] McNairn E., Ni Bhriain N., Dorman C.J. (1995). Overexpression of the Shigella flexneri genes coding for DNA topoisomerase IV compensates for loss of DNA topoisomerase I: Effect on virulence gene expression. Mol. Microbiol..

[34] Suerbaum S., Brauer-Steppkes T., Labigne A., Cameron B., Drlica K. (1998). Topoisomerase I of Helicobacter pylori: Juxtaposition with a flagellin gene (flaB) and functional requirement of a fourth zinc finger motif. Gene.

[35] Ahmed W., Menon S., Godbole A.A., Karthik P.V., Nagaraja V. (2014). Conditional silencing of topoisomerase I gene of Mycobacterium tuberculosis validates its essentiality for cell survival. FEMS Microbiol. Lett..

[36] Liu L.F., Wang J.C. (1987). Supercoiling of the DNA template during transcription. Proc. Natl. Acad. Sci. USA.

[37] Wu H.Y., Shyy S.H., Wang J.C., Liu L.F. (1988). Transcription generates positively and negatively supercoiled domains in the template. Cell.

[38] Rovinskiy N., Agbleke A.A., Chesnokova O., Pang Z., Higgins N.P. (2012). Rates of gyrase supercoiling and transcription elongation control supercoil density in a bacterial chromosome. PLoS Genet..

[39] Sutormin D., Rubanova N., Logacheva M., Ghilarov D., Severinov K. (2019). Single-nucleotide-resolution mapping of DNA gyrase cleavage sites across the *Escherichia coli* genome. Nucleic Acids Res..

[40] Cheng B., Zhu C.X., Ji C., Ahumada A., Tse-Dinh Y.C. (2003). Direct interaction between *Escherichia coli* RNA polymerase and the zinc ribbon domains of DNA topoisomerase I. J. Biol. Chem..

[41] Tiwari P.B., Chapagain P.P., Banda S., Darici Y., Uren A., Tse-Dinh Y.C. (2016). Characterization of molecular interactions between *Escherichia coli* RNA polymerase and topoisomerase I by molecular simulations. FEBS Lett..

[42] Tan K., Zhou Q., Cheng B., Zhang Z., Joachimiak A., Tse-Dinh Y.C. (2015). Structural basis for suppression of hypernegative DNA supercoiling by *E. coli* topoisomerase I. Nucleic Acids Res..

[43] Rui S., Tse-Dinh Y.C. (2003). Topoisomerase function during bacterial responses to environmental challenge. Front. Biosci..

[44] Banda S., Cao N., Tse-Dinh Y.C. (2017). Distinct Mechanism Evolved for Mycobacterial RNA Polymerase and Topoisomerase I Protein-Protein Interaction. J. Mol. Biol..

[45] Ahmed W., Sala C., Hegde S.R., Jha R.K., Cole S.T., Nagaraja V. (2017). Transcription facilitated genome-wide recruitment of topoisomerase I and DNA gyrase. PLoS Genet..

[46] Hraiky C., Raymond M.A., Drolet M. (2000). RNase H overproduction corrects a defect at the level of transcription elongation during rRNA synthesis in the absence of DNA topoisomerase I in *Escherichia coli*. J. Biol. Chem..

[47] Baaklini I., Usongo V., Nolent F., Sanscartier P., Hraiky C., Drlica K., Drolet M. (2008). Hypernegative supercoiling inhibits growth by causing RNA degradation. J. Bacteriol..

[48] Drolet M. (2006). Growth inhibition mediated by excess negative supercoiling: The interplay between transcription elongation, R-loop formation and DNA topology. Mol. Microbiol..

[49] Phoenix P., Raymond M.A., Masse E., Drolet M. (1997). Roles of DNA topoisomerases in the regulation of R-loop formation in vitro. J. Biol. Chem..

[50] Drolet M., Bi X., Liu L.F. (1994). Hypernegative supercoiling of the DNA template during transcription elongation in vitro. J. Biol. Chem..

[51] Roy D., Zhang Z., Lu Z., Hsieh C.L., Lieber M.R. (2010). Competition between the RNA transcript and the nontemplate DNA strand during R-loop formation in vitro: A nick can serve as a strong R-loop initiation site. Mol. Cell. Biol..

[52] Stolz R., Sulthana S., Hartono S.R., Malig M., Benham C.J., Chedin F. (2019). Interplay between DNA sequence and negative superhelicity drives R-loop structures. Proc. Natl. Acad. Sci. USA.

[53] Masse E., Phoenix P., Drolet M. (1997). DNA topoisomerases regulate R-loop formation during transcription of the rrnB operon in *Escherichia coli*. J. Biol. Chem..

[54] Masse E., Drolet M. (1999). *Escherichia coli* DNA topoisomerase I inhibits R-loop formation by relaxing transcription-induced negative supercoiling. J. Biol. Chem..

[55] Masse E., Drolet M. (1999). R-loop-dependent hypernegative supercoiling in *Escherichia coli* topA mutants preferentially occurs at low temperatures and correlates with growth inhibition. J. Mol. Biol..

[56] Roy D., Yu K., Lieber M.R. (2008). Mechanism of R-loop formation at immunoglobulin class switch sequences. Mol. Cell. Biol..

[57] Usongo V., Nolent F., Sanscartier P., Tanguay C., Broccoli S., Baaklini I., Drlica K., Drolet M. (2008). Depletion of RNase HI activity in *Escherichia coli* lacking DNA topoisomerase I leads to defects in DNA supercoiling and segregation. Mol. Microbiol..

[58] Usongo V., Martel M., Balleydier A., Drolet M. (2016). Mutations reducing replication from R-loops suppress the defects of growth, chromosome segregation and DNA supercoiling in cells lacking topoisomerase I and RNase HI activity. DNA Repair (Amst).

[59] Yang Z., Hou Q., Cheng L., Xu W., Hong Y., Li S., Sun Q. (2017). RNase H1 Cooperates with DNA Gyrases to Restrict R-Loops and Maintain Genome Integrity in Arabidopsis Chloroplasts. Plant. Cell.

[60] Lopez C.R., Yang S., Deibler R.W., Ray S.A., Pennington J.M., Digate R.J., Hastings P.J., Rosenberg S.M., Zechiedrich E.L. (2005). A role for topoisomerase III in a recombination pathway alternative to RuvABC. Mol. Microbiol..

[61] Hiasa H., Marians K.J. (1994). Topoisomerase III, but not topoisomerase I, can support nascent chain elongation during theta-type DNA replication. J. Biol. Chem..

[62] Hiasa H., Marians K.J. (1996). Two distinct modes of strand unlinking during theta-type DNA replication. J. Biol. Chem..

[63] Hardy C.D., Crisona N.J., Stone M.D., Cozzarelli N.R. (2004). Disentangling DNA during replication: A tale of two strands. Philos. Trans. R. Soc. Lond B Biol. Sci..

[64] DiGate R.J., Marians K.J. (1989). Molecular cloning and DNA sequence analysis of *Escherichia coli* topB, the gene encoding topoisomerase III. J. Biol. Chem..

[65] Lee C.M., Wang G., Pertsinidis A., Marians K.J. (2019). Topoisomerase III Acts at the Replication Fork To Remove Precatenanes. J. Bacteriol..

[66] Li Z., Hiasa H., Kumar U., DiGate R.J. (1997). The traE gene of plasmid RP4 encodes a homologue of *Escherichia coli* DNA topoisomerase III. J. Biol. Chem..

[67] Li Z., Hiasa H., DiGate R. (2006). Characterization of a unique type IA topoisomerase in Bacillus cereus. Mol. Microbiol..

[68] Usongo V., Tanguay C., Nolent F., Bessong J.E., Drolet M. (2013). Interplay between type 1A topoisomerases and gyrase in chromosome segregation in *Escherichia coli*. J. Bacteriol..

[69] Harmon F.G., DiGate R.J., Kowalczykowski S.C. (1999). RecQ helicase and topoisomerase III comprise a novel DNA strand passage function: A conserved mechanism for control of DNA recombination. Mol. Cell.

[70] Harmon F.G., Brockman J.P., Kowalczykowski S.C. (2003). RecQ helicase stimulates both DNA catenation and changes in DNA topology by topoisomerase III. J. Biol. Chem..

[71] Gangloff S., McDonald J.P., Bendixen C., Arthur L., Rothstein R. (1994). The yeast type I topoisomerase Top3 interacts with Sgs1, a DNA helicase homolog: A potential eukaryotic reverse gyrase. Mol. Cell. Biol..

[72] Wu L., Hickson I.D. (2003). The Bloom’s syndrome helicase suppresses crossing over during homologous recombination. Nature.

[73] Plank J.L., Wu J., Hsieh T.S. (2006). Topoisomerase IIIalpha and Bloom’s helicase can resolve a mobile double Holliday junction substrate through convergent branch migration. Proc. Natl. Acad. Sci. USA.

[74] Cejka P., Plank J.L., Bachrati C.Z., Hickson I.D., Kowalczykowski S.C. (2010). Rmi1 stimulates decatenation of double Holliday junctions during dissolution by Sgs1-Top3. Nat. Struct. Mol. Biol..

[75] Kogoma T. (1997). Stable DNA replication: Interplay between DNA replication, homologous recombination, and transcription. Microbiol. Mol. Biol. Rev..

[76] Drolet M., Brochu J. (2019). R-loop-dependent replication and genomic instability in bacteria. DNA Repair (Amst).

[77] Ogawa T., Pickett G.G., Kogoma T., Kornberg A. (1984). RNase H confers specificity in the dnaA-dependent initiation of replication at the unique origin of the *Escherichia coli* chromosome in vivo and in vitro. Proc. Natl. Acad. Sci. USA.

[78] Kaguni J.M., Kornberg A. (1984). Topoisomerase I confers specificity in enzymatic replication of the *Escherichia coli* chromosomal origin. J. Biol. Chem..

[79] Michel B., Sandler S.J. (2017). Replication Restart in Bacteria. J. Bacteriol..

[80] Kasahara M., Clikeman J.A., Bates D.B., Kogoma T. (2000). RecA protein-dependent R-loop formation in vitro. Genes Dev..

[81] Zaitsev E.N., Kowalczykowski S.C. (2000). A novel pairing process promoted by *Escherichia coli* RecA protein: Inverse DNA and RNA strand exchange. Genes Dev..

[82] Maduike N.Z., Tehranchi A.K., Wang J.D., Kreuzer K.N. (2014). Replication of the *Escherichia coli* chromosome in RNase HI-deficient cells: Multiple initiation regions and fork dynamics. Mol. Microbiol..

[83] Banda S., Tiwari P.B., Darici Y., Tse-Dinh Y.C. (2016). Investigating direct interaction between *Escherichia coli* topoisomerase I and RecA. Gene.

[84] Reckinger A.R., Jeong K.S., Khodursky A.B., Hiasa H. (2007). RecA can stimulate the relaxation activity of topoisomerase I: Molecular basis of topoisomerase-mediated genome-wide transcriptional responses in *Escherichia coli*. Nucleic Acids Res..

[85] Tang S., Wu M.K.Y., Zhang R., Hunter N. (2015). Pervasive and essential roles of the Top3-Rmi1 decatenase orchestrate recombination and facilitate chromosome segregation in meiosis. Mol. Cell.

[86] Fasching C.L., Cejka P., Kowalczykowski S.C., Heyer W.D. (2015). Top3-Rmi1 dissolve Rad51-mediated D loops by a topoisomerase-based mechanism. Mol. Cell.

[87] Kaur H., De Muyt A., Lichten M. (2015). Top3-Rmi1 DNA single-strand decatenase is integral to the formation and resolution of meiotic recombination intermediates. Mol. Cell.

[88] Wahba L., Gore S.K., Koshland D. (2013). The homologous recombination machinery modulates the formation of RNA-DNA hybrids and associated chromosome instability. Elife.

[89] Zhang T., Wallis M., Petrovic V., Challis J., Kalitsis P., Hudson D.F. (2019). Loss of TOP3B leads to increased R-loop formation and genome instability. Open Biol..

[90] Leonard A.C., Mechali M. (2013). DNA replication origins. Cold Spring Harb. Perspect. Biol..

[91] Bell S.P., Kaguni J.M. (2013). Helicase loading at chromosomal origins of replication. Cold Spring Harb. Perspect. Biol..

[92] Nollmann M., Crisona N.J., Arimondo P.B. (2007). Thirty years of *Escherichia coli* DNA gyrase: From in vivo function to single-molecule mechanism. Biochimie.

[93] Johnsen L., Weigel C., von Kries J., Moller M., Skarstad K. (2010). A novel DNA gyrase inhibitor rescues *Escherichia coli* dnaAcos mutant cells from lethal hyperinitiation. J. Antimicrob. Chemother..

[94] Baker T.A., Sekimizu K., Funnell B.E., Kornberg A. (1986). Extensive unwinding of the plasmid template during staged enzymatic initiation of DNA replication from the origin of the *Escherichia coli* chromosome. Cell.

[95] Louarn J., Bouche J.P., Patte J., Louarn J.M. (1984). Genetic inactivation of topoisomerase I suppresses a defect in initiation of chromosome replication in *Escherichia coli*. Mol. Gen. Genet..

[96] Kraemer J.A., Sanderlin A.G., Laub M.T. (2019). The Stringent Response Inhibits DNA Replication Initiation in *E. coli* by Modulating Supercoiling of *oriC*. mBio.

[97] Morafraile E.C., Hanni C., Allen G., Zeisner T., Clarke C., Johnson M.C., Santos M.M., Carroll L., Minchell N.E., Baxter J. (2019). Checkpoint inhibition of origin firing prevents DNA topological stress. Genes Dev..

[98] Minden J.S., Marians K.J. (1986). *Escherichia coli* topoisomerase I can segregate replicating pBR322 daughter DNA molecules in vitro. J. Biol. Chem..

[99] Mundbjerg K., Jorgensen S.W., Fredsoe J., Nielsen I., Pedersen J.M., Bentsen I.B., Lisby M., Bjergbaek L., Andersen A.H. (2015). Top2 and Sgs1-Top3 Act Redundantly to Ensure rDNA Replication Termination. PLoS Genet..

[100] De Septenville A.L., Duigou S., Boubakri H., Michel B. (2012). Replication fork reversal after replication-transcription collision. PLoS Genet..

[101] El Sayyed H., Le Chat L., Lebailly E., Vickridge E., Pages C., Cornet F., Cosentino Lagomarsino M., Espeli O. (2016). Mapping Topoisomerase IV Binding and Activity Sites on the *E. coli* Genome. PLoS Genet..

[102] Crisona N.J., Strick T.R., Bensimon D., Croquette V., Cozzarelli N.R. (2000). Preferential relaxation of positively supercoiled DNA by *E. coli* topoisomerase IV in single-molecule and ensemble measurements. Genes Dev..

[103] Dorman C.J. (2019). DNA supercoiling and transcription in bacteria: A two-way street. BMC Mol. Cell. Biol..

[104] Rani P., Nagaraja V. (2019). Genome-wide mapping of Topoisomerase I activity sites reveal its role in chromosome segregation. Nucleic Acids Res..

[105] Bachar A., Itzhaki E., Gleizer S., Shamshoom M., Milo R., Antonovsky N. (2020). Point mutations in topoisomerase I alter the mutation spectrum in *E. coli* and impact the emergence of drug resistance genotypes. Nucleic Acids Res..

[106] Duggin I.G., Wake R.G., Bell S.D., Hill T.M. (2008). The replication fork trap and termination of chromosome replication. Mol. Microbiol..

